# Complete mitochondrial genome of the recently discovered multivoltine *Graphium* (*Pazala*) *confucius* Hu, Duan & Cotton, 2018 (Lepidoptera: Papilionidae)

**DOI:** 10.1080/23802359.2021.2015269

**Published:** 2021-12-28

**Authors:** Fu-Rong He, Xin Zhang, Shao-Ji Hu

**Affiliations:** a School of Life Sciences, Yunnan University, Kunming, China; b Kunming Youning Biotech Co., Ltd, Kunming, China; c Institute of International Rivers and Eco-security, Yunnan University, Kunming, China; d Yunnan Key Laboratory of International Rivers and Transboundary Eco-security, Yunnan University, Kunming, China

**Keywords:** Mitogenome, protein coding genes, transfer RNA, ribosomal RNA, phylogenetic position

## Abstract

*Graphium* (*Pazala*) *confucius* Hu, Duan & Cotton, 2018 is a recently discovered, wide ranging, multivoltine swordtail butterfly in China and Vietnam. The present study reports the complete mitochondrial genome of this butterfly, which is the fifth mitochondrial genome record for subgenus *Pazala* Moore, 1888. The mitochondrial genome of *G.* (*P.*) *confucius* is circular and 15,212 bp in length, and consists of 37 genes, including 13 PCGs, 22 tRNAs, and two rRNAs. The Bayesian phylogenetic tree containing the focal species and 33 other Papilioninae members clusters *G.* (*P.*) *confucius* with other *Pazala* taxa inside tribe Leptocircini, which agrees with its taxonomic position. The findings of this study added data to the complex subgenus *Pazala* and are beneficial to future understanding and conservation planning of butterfly diversity.

Phylogenetic diversity has been increasingly accepted as a tool in conservation planning (Lu and Bullock 2021). To achieve such meta-analysis, the accumulation of molecular data, especially those of the regionally endemic, recently discovered, and little-known species are crucial (Wang et al. 2020). *Graphium* (*Pazala*) *confucius* Hu, Duan & Cotton, 2018 is among several recently discovered species of the subgenus *Pazala* Moore, 1888 (Lepidoptera: Papilionidae), a complex Sino-Himalayan group of butterflies (Racheli and Cotton 2009). *G.* (*P.*) *confucius* occupies a wide distribution range from Southwest to Central China, as well as North Vietnam (Hu et al. 2018); and it is also the only known multivoltine *Pazala* species with three generations per year (Zhang et al. 2018). The mitochondrial genome of *G.* (*P.*) *confucius* reported herein would add new data to facilitate future understanding and conservation planning for butterflies.

The butterfly specimen used in this study was collected from Da Moyu (25.066411°N, 102.589607°E, 2200 m), Kunming, Yunnan, China. The specimen was deposited in the Zoological Museum (insect collection) of Yunnan University, Kunming, China (specimen number: YNU-LEP-PAP-2021GC01, contact person: Shao-Ji Hu). Genomic DNA was extracted from the thoracic muscle tissue of a single male adult using the TianGen TIANamp Genome DNA Kit (TianGen Biotech Co., Ltd., Beijing, China). The PCR amplification was performed using in a 25 μl system containing 2.5 μl 10× PCR buffer, 2 μL MgCl_2_ (25 mM), 2 μl dNTPs (2.5 mM each), 0.5 μl each of forward and reverse primers (20 μM; Table S1), 0.25 μl *Taq* DNA polymerase (TaKaRa Biotechnology Co., Ltd., Dalian, China), and 1 μl genomic DNA. The PCR thermal profile consisted of an initially denaturation at 95 °C for 3 min; followed by 30 cycles of 94 °C denaturation for 60 s, 50 °C annealing for 60 s, and 72 °C extension for 90 s; then an external extension at 72 °C for 5 min. All PCR products were sequenced on an ABI 3730xl automatic sequencer (Applied Biosystems, CA, USA). Resultant gene fragments were assembled using DNAStar (https://www.dnastar.com/) with *G.* (*P.*) *parus* (MT198821), *G.* (*P.*) *mullah chungianus* (MW549197), and *G.* (*P.*) *eurous asakurae* (MW549198) as the reference genomes (Duan et al. 2020; Hu et al. 2021). Transfer RNA genes (tRNAs) and ribosomal RNA genes (rRNAs) were predicted using the web based MITOS (http://mitos.bioinf.uni-leipzig.de/index.py) (Bernt et al. 2013), while all PCGs were determined using the Alignments | CDS feature under BLASTn of NCBI (https://blast.ncbi.nlm.nih.gov/).

The complete mitochondrial genome of *G.* (*P.*) *confucius* is circular and 15,212 bp in length (GenBank accession number: OK136253). The base composition is 39.84% for A, 39.28% for T, 8.13% for G, and 12.75% for C. This mitochondrial genome contains 37 genes, including 13 PCGs, 22 tRNAs, and two rRNAs, plus a non-coding control region. The plus (+) strand encodes nine PCGs (*nad2*, *cox1*, *cox2*, *atp8*, *atp6*, *cox3*, *nad3*, *nad6*, and *cob*), while the minus (−) strand encodes four PCGs (*nad5*, *nad4*, *nad4l*, and *nad1*).The gene arrangement and character of this genome fit those of ditrysian Lepidoptera mitochondrial genomes (Cao et al. 2012; Chen et al. 2020; Wang et al. 2019).

To validate this mitochondrial genome, a Bayesian phylogenetic tree was reconstructed by PhyloSuite 1.2.2 (Zhang et al. 2020) using the 37 genes (13 PCGs, 22 tRNAs, and two rRNAs) for 1,000,000 generations, with the GTR + F + I + G model selected by ModelFinder (Kalyaanamoorthy et al. 2017). Thirty-three species of Papilioninae with available mitochondrial genomes were used as ingroups and *Parnassius apollo* Linnaeus, 1758 (Parnassiinae; KF746065) (Chen et al. 2014) was chosen as the outgroup. The result shows that *G.* (*P.*) *confucius* clusters with *G.* (*P.*) *parus* first and then with other *Pazala* species. All *Pazala* taxa are related to other *Graphium* species within Leptocircini, forming a monophyletic clade, supported by the maximal support values (Figure 1).

**Figure 1.**
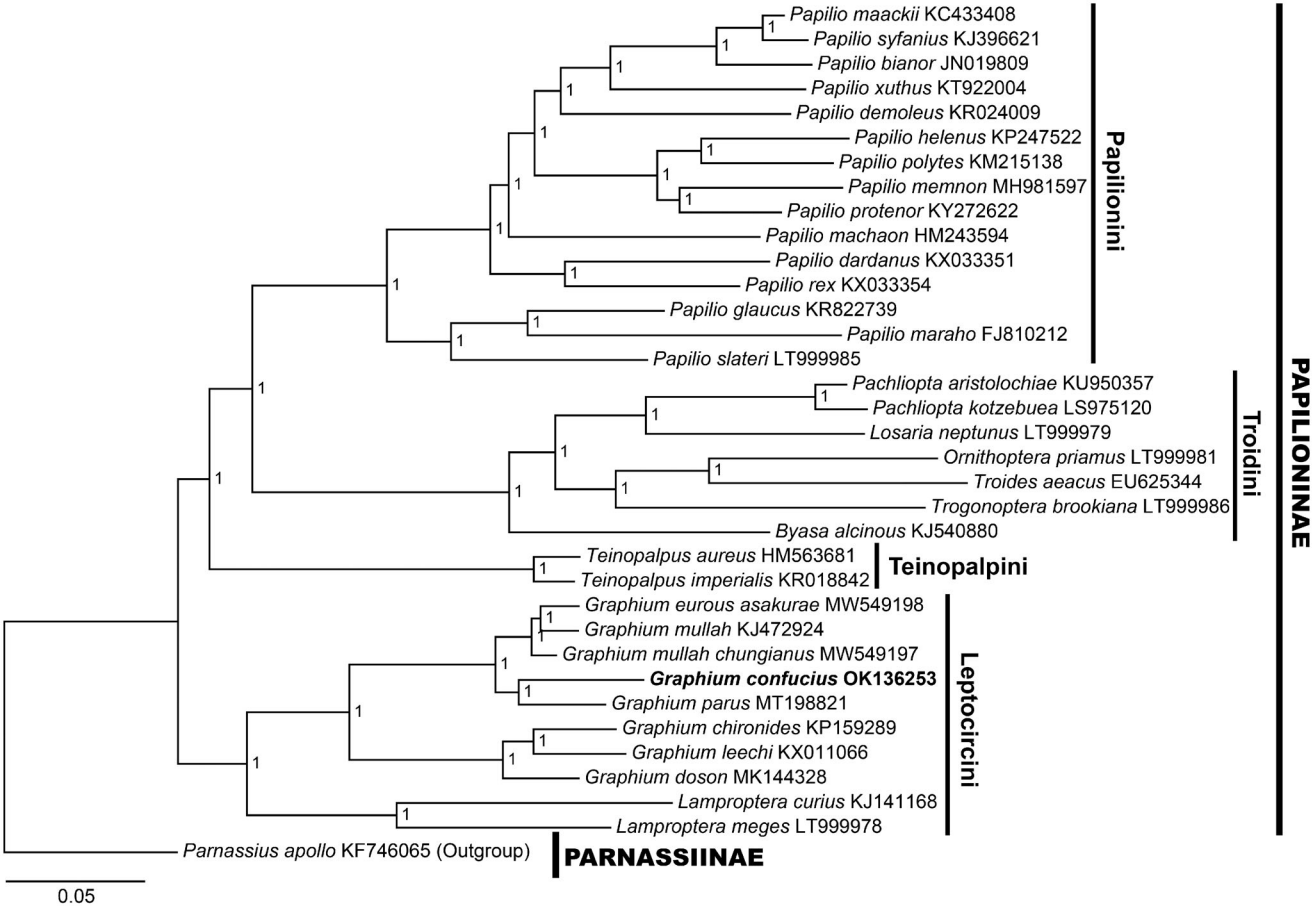
The Bayesian phylogenetic tree for *Graphium* (*Pazala*) *confucius* Hu, Duan & Cotton, 2018 (marked with bold font) and other Papilioninae taxa. Node labels represent support values.

## Supplementary Material

Supplemental Material

## Data Availability

The data supporting the findings of this study is openly available in the NCBI GenBank at https://www.ncbi.nlm.nih.gov/genbank/, accession numbers of all used sequences are listed in Figure 1.

## References

[CIT0001] Bernt M, Donath A, Jühling F, Externbrink F, Florentz C, Fritzsch G, Pütz J, Middendorf M, Stadler PF. 2013. MITOS: improved *de novo* metazoan mitochondrial genome annotation. Mol Phylogenet Evol. 69 (2):313–319. 22982435 10.1016/j.ympev.2012.08.023

[CIT0002] Cao YQ, Ma C, Chen JY, Yang DR. 2012. The complete mitochondrial genomes of two ghost moths, *Thitarodes renzhiensis* and *Thitarodes yunnanensis*: the ancestral gene arrangement in Lepidoptera. BMC Genomics. 13:276. 22726496 10.1186/1471-2164-13-276 PMC3463433

[CIT0003] Chen YH, Huang DY, Wang YL, Zhu CD, Hao JS. 2014. The complete mitochondrial genome of the endangered Apollo butterfly, *Parnassius apollo* (Lepidoptera: Papilionidae) and its comparison to other Papilionidae species. J Asia-Pac Entomol. 17(4):663–671.

[CIT0004] Chen L, Wahlberg N, Liao CQ, Wang CB, Ma FZ, Huang GH. 2020. Fourteen complete mitochondrial genomes of butterflies from the genus *Lethe* (Lepidoptera, Nymphalidae, Satyrinae) with mitogenome-based phylogenetic analysis. Genomics. 112(6):4435–4441. 32745503 10.1016/j.ygeno.2020.07.042

[CIT0005] Duan K, Zhang X, Zhang HH, Hu SJ. 2020. Complete mitochondrial genome of the subalpine swordtail butterfly *Graphium* (*Pazala*) *parus* (Nicéville, 1900) (Lepidoptera: Papilionidae). Mitochondrial DNA Part B. 5(2):1903–1904.

[CIT0006] Hu SJ, Cotton AM, Condamine FL, Duan K, Wang RJ, Hsu YF, Zhang X, Cao J. 2018. Revision of *Pazala* Moore, 1888: the *Graphium* (*Pazala*) *mandarinus* (Oberthür, 1879) group, with treatments of known taxa and descriptions of new species and new subspecies (Lepidoptera: Papilionidae). Zootaxa. 4441 (3):401–446. 30313994 10.11646/zootaxa.4441.3.1

[CIT0007] Hu SJ, Zhang X, Duan K. 2021. Complete mitochondrial genomes of two insular races of *Pazala* swordtails from Taiwan, China (Lepidoptera: Papilionidae: *Graphium*). Mitochondrial DNA Part B. 6(4):1557–1559. 33969217 10.1080/23802359.2021.1915719 PMC8079028

[CIT0008] Kalyaanamoorthy S, Minh BQ, Wong TKF, von Haeseler A, Jermiin LS. 2017. ModelFinder: fast model selection for accurate phylogenetic estimates. Nat Methods. 14(6):587–589. 28481363 10.1038/nmeth.4285 PMC5453245

[CIT0009] Lu YH, Bullock JM. 2021. Biodiversity conservation in a changing environment beyond 2020. Sci Advance. 7:eabl8162. 10.1126/sciadv.abl8162 PMC11559486 34433572

[CIT0010] Racheli T, Cotton AM. 2009. Guide to the butterflies of the Palearctic region. Papilionidae. Part I. Milano: Omnes Artes.

[CIT0011] Wang X, Chen ZM, Gu XS, Wang M, Huang GH, Zwick A. 2019. Phylogenetic relationships among Bombycidae s.l. (Lepidoptera) based on analyses of complete mitochondrial genomes. Syst Entomol. 44(3):490–498.

[CIT0012] Wang WL, Suman DO, Zhang HH, Xu ZB, Ma FZ, Hu SJ. 2020. Butterfly conservation in China: from science to action. Insects. 11(10):661. 10.3390/insects11100661 PMC7600441 32992975

[CIT0013] Zhang HH, Duan K, Hu SJ. 2018. On the immature stages of *Graphium* (*Pazala*) *confucius* Hu, Duan & Cotton, 2018 observed in Kunming, Yunnan, China. Butterflies. 79:18–25.

[CIT0014] Zhang D, Gao FL, Jakovlić I, Zou H, Zhang J, Li WX, Wang GT. 2020. PhyloSuite: an integrated and scalable desktop platform for streamlined molecular sequence data management and evolutionary phylogenetics studies. Mol Ecol Resour. 20(1):348–355. 31599058 10.1111/1755-0998.13096

