# Supplementary material for: Complete mitochondrial genome of the recently discovered multivoltine Graphium (Pazala) confucius Hu, Duan & Cotton, 2018 (Lepidoptera: Papilionidae)
Source: Mitochondrial DNA B Resour. 2021 Dec 28;7(1):138–40. doi: 10.1080/23802359.2021.2015269 (PMC8725947; doi:10.1080/23802359.2021.2015269)
Supplement: Supplemental Material [file TMDN_A_2015269_SM6138.docx]

**Table S1. Primers used for PCR amplification of the mitochondrial genome of *Graphium* (*Pazala*) *confucius* Hu, Duan & Cotton, 2018**

| **Primer** | **Nucleotide sequence (5' to 3')** |
| --- | --- |
| F1 | TTAAAAATAAGCTAAATRAAGCTTTT |
| R648 | GGTGAAATTTTTTGTCAKGTTATAAT |
| F542 | TTAATAAAAATAGGATCAGCCCCCTTT |
| R1432 | ATGGCTGAGTTTAAGCGATAAATTGTA |
| F1297 | AGAAATTCTTTAAGCCTTAGTATTTTA |
| R1894 | CTGCTAAATGRAGGGAAAAAATAGC |
| F1762 | ATYCTCYTAATTTCAAGAAGAATTG |
| R2430 | AATTTGAGTTCCATGTAAAGTTGCTA |
| F2269 | GCAATTGGYCTTTTAGGATTTATTGTA |
| R3058 | TATATCAAGTTGCCATTTCTAAAAAA |
| F2952 | ATGAACTYCCTATTTTAAGAAACTTCT |
| R3667 | TAGGTATGAAKCTATGATTAGCTCCA |
| F3550 | RTTAAAATTGAYGCAAATCCTGGTC |
| R4239 | TAAATGTTGATCCATTAAATCTATTAGG |
| F4048 | TGACCCCTCAACTAATATTTTTAACT |
| R4746 | YGGYCTATAATCAACTAAGTGAAATG |
| F4617 | TAGTATTAGAATCAGCAGTAGCTATTAT |
| R5302 | TAGCYATAAAAAATGTTGATCCATAA |
| F5131 | GTAACYTGAGCTCAYCATGCTATTATA |
| R5780 | AAAATTAATGCRATTTCAATATCAA |
| F5630 | TTATCAAAAAAATCATTTATAGACCG |
| R6444 | AATTRTATAGAGGTCAAGGTATATTTAA |
| F6240 | AAGATTATTYAATCTCCCTAATATCTTC |
| R6963 | TAGCRGGATTTTATTCTAAGGATTTA |
| F6862 | TAGTTAAACCYGTAGAAAAATAATATA |
| R7615 | ATATTAACTGCTTTATCTAATCGAATT |
| F7422 | CAAGAACTAAAAGGAATYTGAGCACT |
| R8080 | TTTGTGGAGTCAAAAATATGATAA |
| F7806 | CTRTAATAAATWACTGAAGATGAAATT |
| R8619 | TTCTGTYGCTCAYATAAGATTAGTAA |
| F8505 | AAATATYCCAGAAGAACATAATCCAT |
| R9108 | ATTTTTTGAGGGGAGATTAATTCCTA |
| F9021 | ATAAATATAAACCAGCTTGAATTCG |
| R9783 | GRTTTACAAGACCAATGTTTTATT |
| F9643 | AATAAAACAATTAATAAATGYTTATGTTTAG |
| R10541 | TAAACCTARTAAAGAGCCAAAATTTCA |
| F10385 | GGRCCYTTACGATCTATAAATTAAAC |
| R11055 | CCTAAWGGRTTRTTAGATCCTGTTTG |
| F10934 | AGATAAYGCYACATTRACACGATTTTA |
| R11724 | TCTTGAAAAAGATATTTACCTGTTTCTT |
| F11565 | TTAATTAATGAGCTTGTRAAAGCAT |
| R12345 | CCTGTAGTTGGGTTTATTTTATCTTTAA |
| F12140 | TCATAAGAAATAGTTTGAGCTACAGC |
| R12925 | TAAATTACCTTAGGGATAACAGCGTAA |
| F12784 | ATGTAAGATTTTAATGATCGAACAGA |
| R13274 | TGGTCTGCCCACTGATAATATATT |
| F13175 | AATCYTTCATACAAGTCACCAATT |
| R13821 | TTGTATCTTGTGTATCAGAGTTTATTAA |
| F13684 | AACGATTAACATTTCATTTCCAATTA |
| R14408 | GGAATCTGTCTAATAATTGATAATCC |
| F14260 | TTAATTATTATCTGCATCTTGATCTGA |
| F14635 | ATTATATTTTGTATAACCGCAACTG |
| R14635 | CAGTTGCGGTTATACAAAATATAAT |
| F14846 | CACATAGAATTTTTTTTTTTTTTT |
| R14846 | AAAAAAAAAAAAAAATTCTATGTG |
| R102 | TCTATCARAATAATCCTTTAATCAGGC |
